# Exploring the Current Trends of Artificial Intelligence in Stem Cell Therapy: A Systematic Review

**DOI:** 10.7759/cureus.20083

**Published:** 2021-12-01

**Authors:** Mirra Srinivasan, Santhosh Raja Thangaraj, Krishnamurthy Ramasubramanian, Padma Pradha Thangaraj, Krishna Vyas Ramasubramanian

**Affiliations:** 1 Internal Medicine, California Institute of Behavioral Neurosciences and Psychology, Fairfield, USA; 2 Internal Medicine, Rajah Muthiah Medical College, Chidambaram, IND; 3 Computer Science and Engineering, Koneru Lakshmaiah University, Koneru Lakshmaiah Education Foundation (KLEF), Hyderabad, IND; 4 Instrumentation Engineering, Madras Institute of Technology, Anna University, Chennai, IND; 5 Computer Science and Engineering, Artificial Intelligence and Machine Learning, Vellore Institute of Technology, Chennai, IND

**Keywords:** induced pluripotent stem cells, deep neural network, machine learning, artificial intelligence, stem cell transplantation, therapeutic uses, stem cell therapy

## Abstract

The concept of *healing in medicine* has been taking a new form where scientists and researchers are in pursuance of regenerative medicine. Until now, doctors have "reacted" to disease by treating the symptoms; however, modern medicine is transforming toward regeneration rather than reactive treatment, which is where stem cell therapy comes into the play-the concept of replacing damaged cells with brand new cells that perform the same function better. Stem cell treatment is currently being used to treat autoimmune, inflammatory, neurological, orthopedic, and traumatic disorders, with various research being undertaken for a wide range of diseases. It could also be the answer to anti-aging and a disease-free state. Despite the benefits, numerous errors could prevail in treating patients with stem cells. With the advancement of technology and research in the modern period, medicine is beginning to turn to artificial intelligence (AI) to address the complicated errors that could occur in regenerative medicine. For successful treatment, one must achieve precision and accuracy when analyzing healthy and productive stem cells that possess all the properties of a native cell. This review intends to discuss and study the application of AI in stem cell therapy and how it influences how medicine is practiced, thus creating a path to a regenerative future with negligible adverse effects.

The following databases were used for a literature search: PubMed, Google Scholar, PubMed Central, and Institute of Electrical and Electronics Engineers (IEEE) Xplore. After a thorough analysis, studies were chosen, keeping in mind the inclusion and exclusion criteria set by the authors of this review, which comprised reports published within the last six years in the English language. The authors also made sure to include studies that sufficed the quality of each report assessed using appropriate quality appraisal tools, after which eight reports were found to be eligible and were included in this review. This research mainly revolves around machine learning, deep neural networks (DNN), and other subclasses of AI encompassed in these categories.

While there are concerns and limitations in implementing various mediums of AI in stem cell therapy, the analysis of the eligible studies concluded that artificial intelligence provides significant benefits to the global healthcare ecosystem in numerous ways, such as determining the viability, functionality, biosafety, and bioefficacy of stem cells, as well as appropriate patient selection. Applying AI to this novelty brings out the precision, accuracy, and a revolution in regenerative medicine. In addition, stem cell therapy is not currently FDA approved (except for the blood-forming stem cells) and, to date, is considered experimental with no clear outline of risks and benefits. Given this limitation, studies are conducted regularly around the world in hopes for a concrete conclusion where technological advances such as AI could help in shaping the future of regenerative medicine.

## Introduction and background

At a symposium at Dartmouth in 1956, computer scientist John McCarthy defined the term "Artificial Intelligence" (AI) as "the science and engineering of making intelligent machines, especially intelligent computer programs" [1]. As technological advances are happening every day, the evolution of AI has also taken its place in the scientific world, where the purpose of AI is to create machines that can think, reason, see, and hear like a human to rise beyond human intelligence [2]. Artificial intelligence allows computers to learn, reason, and achieve goals [3] with the least possible human intervention [4]. Machine learning is considered a division of AI that enables machines to comprehend and remember large amounts of data without being distinctly programmed [3].

AI was developed, in an aspiration, to reach every area of human activity in order to lessen people's efforts associated with mundane daily tasks as well as complex tasks with an endeavor to achieve accuracy and precision [5]. AI has been incorporated in limited technologies resembling human intelligence in some cognitive areas such as speech and facial recognition [2]. Nowadays, machine learning (ML) and deep learning (DL) have advanced AI in various domains, including image classification, text analysis, speech/facial recognition, autonomous automobiles, natural language processing, and also in medicine [2]. The capability of analyzing complex medical data and the potential to utilize meaningful connections within a dataset can not only be used in the diagnosis and treatment but also can be used in predicting outcomes in several clinical scenarios [6], thus affirming the role of AI to be significant in healthcare [2]. The emergence of complex illnesses has created many opportunities to leverage technology to deploy more explicit, efficient, and impactful interventions to patient care at precisely the right moment [7].

According to the report of Frost and Sullivan derived from the Artificial Intelligence and Cognitive Computing Systems in Healthcare, the industry earned $633.8 million in 2014 and is expected to reach $6.6 billion by 2021 at a 40% annual growth rate. Overall, AI has the potential to enhance outcomes by 30% to 40% while reducing treatment costs by up to 50% [8].

Regenerative medicine is gaining more and more popularity in today's era, and the research on stem cell treatment has shown that stem cells or their products can enhance the repair response of any diseased, damaged, or wounded tissue. It is the next step in organ transplantation because it employs cells rather than donor organs, which are in short supply [9]. In the United States, the only FDA-approved stem cell products for use are blood-forming stem cells (also known as hematopoietic progenitor cells) obtained from umbilical cord blood [10]. Numerous researches are aiming to steer for successful, safe, and approved treatments using stem cells in a wide range of ailments. Companies such as Biotech Cellino are investing $75 million in an effort to merge AI technology in the development of automated stem cell manufacturing, which has the potential to democratize access to cell treatments while also trying to be cost-effective [11]. The purpose of this review is to study the advances and various methods of implementing AI in stem cell therapy as well as to enumerate the merits and demerits of employing AI in the field of regenerative medicine.

Methodology

The Preferred Reporting Items for Systematic Reviews and Meta-Analysis (PRISMA) guidelines 2020 and principles were followed in this systematic review [12].

Inclusion and Exclusion Criteria

The inclusion criteria for this study are set as full-text papers in the English language published in the last six years (2015-2021), with a global search of results including review articles, observational studies, comparative studies, animal studies, experimental studies, and government publications. The non-English, non-full text articles and studies focusing on treatment modalities other than stem cell therapy were excluded. The population, intervention, comparison, outcome, and study criteria (PICOS) were incorporated in this study with the population group consisting of adults (female and male) and animals.

Information Sources and Search Strategy

A total of 247 articles were selected with a literature search done in four databases, namely, PubMed, Google Scholar, PubMed Central (PMC), and Institute of Electrical and Electronics Engineers (IEEE) Xplore. The relevant articles were acquired electronically using keywords in a Boolean scheme, including the MeSH keywords employed in PubMed, as listed below. The articles retrieved were checked in-depth for the titles, abstracts, subject headings, and references, thus excluding all irrelevant reports.

Keywords

MeSH Keywords in PubMed included: Stem cells OR regenerative medicine OR ("Stem Cells"[Mesh] OR "Stem Cell Research"[Mesh]) AND artificial intelligence OR machine learning OR knowledge engineering OR ("Artificial Intelligence/classification"[Mesh] OR "Artificial Intelligence/ethics"[Mesh] OR "Artificial Intelligence/instrumentation"[Mesh] OR "Artificial Intelligence/methods"[Mesh]).

Keywords on other databases: Stem cell therapy, therapeutic uses, stem cell transplantation, artificial intelligence, machine learning (ML), deep neural network (DNN), induced pluripotent stem cells.

Data Extraction and Selection Process

The study team employed relevant quality assessment techniques during the selection process to determine if the studies matched the inclusion criteria. The data selection and extraction were carried out by two researchers independent of one another. Data were collected and extracted from the four databases mentioned above between October 10, 2021, and October 25, 2021. In instances of dispute, both researchers discussed the study designs, inclusion and exclusion criteria, intervention employed, and outcome measured to reach an agreement. In equivocal events, a third reviewer was consulted to settle disagreements to attain a common ground.

Quality Assessment

Table 1 below depicts the type of study reviewed and corresponding scores awarded to each study according to the respective quality appraisal tools [13-16].

**Table 1:** Quality Appraisal Tools and Scores Given for the Final Studies

Type of study	Number of studies	Quality appraisal tool	Scores
Animal study [13]	3	SYRCLE's assessment tool	Low risk of bias
Cohort [14]	1	Newcastle-Ottawa	>10
Review article [15]	1	SANRA Checklist	>9
Quasi-experimental [16]	3	JBI critical appraisal checklist	Overall appraisal: include (>7)

Results

**Figure 1:**
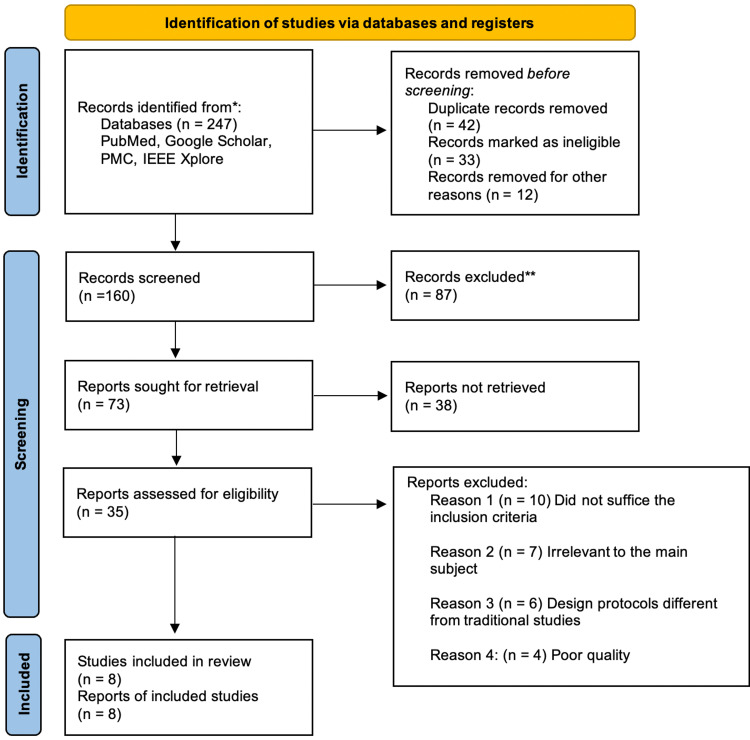
PRISMA Flow Diagram

As mentioned above, the search method applied in this investigation encompassed four separate databases. This search generated 247 articles, out of which 42 were duplicates and thus eliminated manually, 33 of which were removed due to ineligible entries, 12 were removed for other reasons, and no automated techniques were employed. A total of 160 records were screened, with 87 being eliminated due to irrelevancy based on inclusion/exclusion criteria. Out of 73 articles that were sought for retrieval, 38 reports could not be retrieved due to inadequate study subjects, and unavailability of full-text articles, reducing the final screening to 35 reports that were reviewed for quality and eligibility. After a detailed evaluation, the definitive studies considered for this review were eight reports. Figure 1 depicts the search technique employed in the PRISMA flow diagram 2020 format below [12].

The summary of the studies, along with the results, is illustrated in Table 2.

**Table 2:** Summary of All Studies With Findings and Conclusion

Shouval et al. [17]		
Title	Prediction of Allogeneic Hematopoietic Stem-Cell Transplantation (HSCT) Mortality 100 Days After Transplantation Using a Machine Learning Algorithm: A European Group for Blood and Marrow Transplantation Acute Leukemia Working Party Retrospective Data Mining Study.	
Type of Study; Number of Subjects	Retrospective cohort; 28,236	
Purpose	Acute leukemia can be cured by allogeneic hematopoietic stem-cell transplantation; however, the adverse effects of this therapy are to be considered. Machine learning methods, part of the data mining (DM) methodology, may help predict transplant-related mortality risk.	
Findings/Results	On day 100, the prevalence of overall mortality (OM) was 13.9%. The model analyzed ten variables, and the crude score was adjusted for 100-day OM according to individual probabilities using a logistic transformation method (range, 3% to 68%). The primary outcome determined by the model outperformed the European Group for Blood and Marrow Transplantation score (area under the receiver operating characteristic curve, 0.701 vs 0.646; P=0.001). The calibration was considered flawless, and the secondary objectives were also predicted according to the allocated scores.	
Conclusion	The data mining method proved to help predict 100-day overall mortality and has extended that prediction to up to two years. This tool is available online to aid in the risk evaluation of patients with acute leukemia before hematopoietic stem cell therapy.	
Fan et al. [18]		
Title	A Machine Learning Assisted, Label-free, Noninvasive Approach for Somatic Reprogramming in Induced Pluripotent Stem Cell Colony Formation Detection and Prediction	
Type of Study	Animal study	
Purpose	A time-lapse-based brightfield imaging analysis system was studied in order to implement a label-free, noninvasive approach to measure morphological dynamics in the desire to reduce errors, especially when done manually by humans. In order to analyze and identify iPSC colony formation in an automated manner, a machine learning-based classification, segmentation, and statistical modeling method were built to facilitate colony selection.	
Findings/Results	The findings suggested that the system discovered and projected colonies with no significant variations in biological characteristics (Pearson coefficient r > 0.9) compared to manually processed colonies. This was tested and defined physiologically for pluripotency confirmation using conventional immunofluorescence labeling, quantitative polymerase chain reaction (QPCR), and RNA-Seq.	
Conclusion	In terms of biological properties, these algorithm-detected colonies exhibit no significant differences compared to manually processed colonies using traditional molecular techniques.	
Kavita et al. [19]		
Title		Deep Vector-Based Convolutional Neural Network Approach for Automatic Recognition of Colonies of Induced Pluripotent Stem Cells
Type of Study		Animal study (murine embryonic fibroblasts)
Purpose		To evaluate whether the vector-based convolutional neural network (V-CNN) is the preferred model for recognizing colony quality from morphological and textural features of a segmented colony than support vector machine (SVM), while also verifying results of colony quality recognition using an exact cross-validation process, as well as to exhibit the superiority of the proposed deep V-CNN learning approach over the SVM classification.
Findings/Results		The V-CNN model was compared to SVM in differentiating colonies classifier based on morphological, textural, and combination data. In the process of identifying the quality of colonies, the V-CNN model outperformed the SVM classifier in terms of morphological (95.5%), textural (91.0%), and combined (93.2%), accuracy (86.7%, 83.3%, and 83.4%, respectively).
Conclusion		The suggested V-CNN model outperforms the classic SVM classifier, meaning it is a reliable framework for iPSC colony classification while also functioning as a cost-effective quality recognition tool during culture and other experimental procedures.
Waisman et al. [20]		
Title		Deep Learning Neural Networks Highly Predict Very Early Onset of Pluripotent Stem Cell Differentiation
Type of Study		Quasi-experiment
Purpose		The purpose was to attain accuracy in distinguishing pluripotent stem cells from early differentiating cells by applying the concept of convolutional neural networks (CNNs), a branch of deep learning, by using transmitted light microscopy images.
Findings/Results		Mouse embryonic stem cells were stimulated to differentiate into epiblast-like cells and photographed at various time points after the first stimulation. The study observed that the networks could be taught to distinguish between undifferentiated and differentiating cells with more than 99% accuracy in 20 minutes.
Conclusion		Accurate cellular morphology identification in a basic microscopic setup might significantly influence how cell tests are done in the future.
Schaub et al. [21]		
Title		Deep Learning Predicts Function of Live Retinal Pigment Epithelium from Quantitative Microscopy
Type of Study		Quasi-experiment
Purpose		To validate transplant function in clinical biomanufacturing - a reliable and noninvasive method was experimented with to predict tissue function and cellular donor identity.
Findings/Results		Noninvasive quantitative brightfield absorbance microscopy (QBAM) imaging may be utilized to examine the pigmentation development of healthy and sick induced pluripotent stem cell-derived retinal pigment epithelial cells (iPSC-RPE). DNNs can assess these pictures and predict cell transepithelial resistance (TER) and vascular endothelial growth factor (VEGF) ratio across 10 different iPSC-RPE preparations. At the same time, QBAM enables DNNs to segment cell boundaries of live RPE cells reliably. QBAM pictures include enough information to calculate hundreds of characteristics per cell, and these features may be used to forecast cell function, identify outlier samples, and authenticate donor identification. All of this information may be collected in minutes using an automated brightfield microscope on the tissue that is being transplanted.
Conclusion		These findings show that noninvasive cell therapy characterization is possible using QBAM and machine learning.
Aida et al. [22]		
Title		Deep Learning of Cancer Stem Cell Morphology Using Conditional Generative Adversarial Networks
Type of Study		Animal study
Purpose		Cancer stem cells (CSCs) have been essentially characterized by stem cell (SC)-like gene expression with an ability to progress to tumor formation, although the morphological depiction remains unclear. The purpose of this research is to explore the segmentation of CSCs in phase-contrast imaging using conditional generative adversarial networks (CGAN).
Findings/Results		AI was trained using fluorescence images of the Nanog-Green fluorescent protein, which was found to be expressed in CSCs, along with phase-contrast images. The AI model segregated the CSC territory in the phase-contrast images of the CSC cultures and tumor model. Several values for assessing segmentation quality increased when images were chosen for training. Furthermore, nucleus fluorescence overlaid-phase contrast increased the values.
Conclusion		The CGAN-based deep-learning system might be effective in identifying undescribed morphological traits in CSCs.
Juhola et al. [23]		
Title		Analysis of Drug Effects on iPSC Cardiomyocytes with Machine Learning
Type of Study		Quasi-experiment (clinical trial)
Purpose		This study uses machine learning methods to analyze the calcium transient signals and pharmacological effects of induced pluripotent stem cell-derived cardiomyocytes (iPSC-CMs).
Findings/Results		The effects of six iPSC lines containing various mutations that cause a highly malignant hereditary arrhythmogenic condition, catecholaminergic polymorphic ventricular tachycardia (CPVT), were studied. The best classification accuracy was around 79%, demonstrating that machine learning approaches may be used to analyze iPSC-CM medication effects.
Conclusion		The study concluded that machine learning could anticipate the drug effect with high accuracy.
Zaman et al. [24]		
Title		Machine Learning in Stem Cells Research: Application for Biosafety and Bioefficacy Assessment
Type of Study		Review article
Purpose		This study aims to analyze the biosafety and bioefficacy concerns of stem cells for clinical application using machine learning, with a focus on assessing the detrimental effect of tumorigenesis associated with stem cell therapy.
Findings/Results		The model developed might potentially be used to discover fundamental design principles for creating an appropriate microenvironment for stem cell development without compromising their mortality or modifying their epigenetic components, in turn preventing cellular abnormalities.
Conclusion		With the appropriate machine learning and deep learning models, one can assess both biosafety and bioefficacy of stem cells for clinical application.

## Review

This section of the review focuses on explaining stem cell therapy, the application of artificial intelligence in health care, and an in-depth analysis of AI in stem cell therapy. The limitations of this review are also discussed in this section.

What exactly is stem cell therapy?

Stem cells are defined as the "seed" for any living being to develop [25]. These cells possess self-renewal properties and can even differentiate into specific cells of the body when and where required [26]. There are two prominent stem cells types: the pluripotent stem cells (PSC), also known as (a.k.a) embryonic stem cells, and the multipotent a.k.a adult stem cells [27].

**Figure 2:**
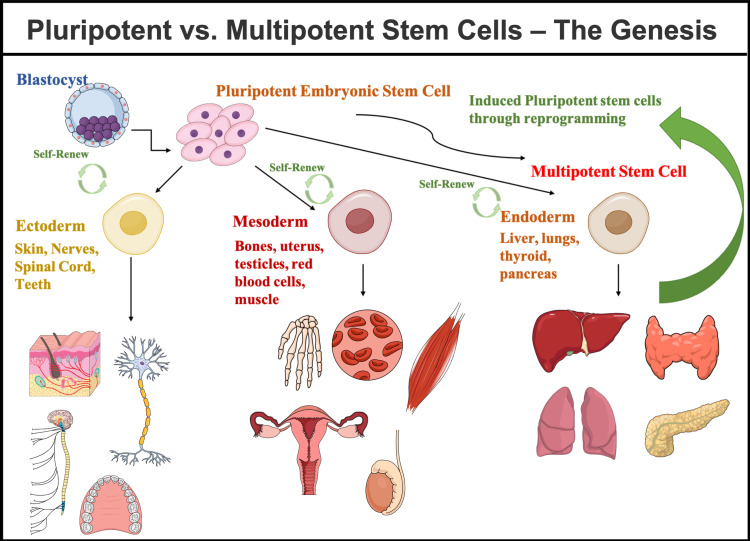
The Genesis of Pluripotent and Multipotent Stem Cells Figure created in Mind the Graph platform

Life begins by the genesis of a zygote - a single cell formed during the fertilization process. This zygote further undergoes replication and further develops into embryonic cells [28]. These embryonic cells serve as the precursor for developing other cells in the body, namely nerve, muscle, blood, etc., thus proving that the pluripotent cells can transform into any cell in an adult body [28]. Pluripotent cells can also develop into multipotent cells that are more specialized to the tissue or organ where they are terminally differentiated [26]. A multipotent blood stem cell, for example, can differentiate into red blood cells, white blood cells, or platelets (all specialized cells) [28]. The main function of these specialized cells is to aid in the repair of any damage caused to the body and pave the way for a regenerative healing process [27]. Figure 2 depicts the origin and development of stem cells into various cells, tissues, and organs in the body [29].

Stem cell therapy dates back to the late 1950s with the story of successful allogeneic bone marrow transplants in scientists who were accidentally exposed to radiation [30]. Following this event, numerous developments were made in treating patients with stem cells. In the 1960s, a breakthrough discovery was made where scientist John Gurdon cloned frogs from somatic cells [31]. This experiment severed the idea that pluripotent cells were only capable of a unidirectional differentiation and bought about the notion that somatic cells, too, could acquire a pluripotent state [31]. One other study worth mentioning was the recognition of reprogramming cells through the process of fibroblast DNA subtraction by Davis et al. The conversion of fibroblasts to myoblasts occurred primarily due to the expression of the myogenic differentiation (Myod1) gene [32]. This study showed that cells could be transformed from one lineage to another. Now that earlier studies have established a basic concept of stem cells, and their uses, more and more studies are emerging to predict the actual fate of these pluripotent and multipotent stem cells and how they work with various ailments.

Pluripotent stem cell therapy

Numerous animal studies have shown promising results where pluripotent stem cell therapy has attempted to cure illnesses like diabetes, acute spinal cord injury, visual impairment, etc. [26,33]. The injected pluripotent cells generated insulin-producing cells, myelinated neurons, and retinal epithelial cells for the abovementioned illness. However, the early studies where scientists could not limit the proliferation capacity resulted in the unwanted formation of solid tumors due to a mix of cell types found in the early germ layers. Due to this hindrance, human trials have not been done with pluripotent stem cells [26]. In attempts to further enhance the benefits of pluripotent stem cells, scientists have evidenced improvement in cardiac function in damaged rodent hearts after injecting them with human cardiac myocytes derived from embryonic stem cells [34]. The reason for this improved function is yet to be fully understood, and a definite conclusion could not be made when it comes to regenerated heart cells.

Multipotent stem cell therapy

In the early 1960s, Ernest McCulloch and James Till worked on experiments in mice to discover the formation of blood cells through the hematopoietic stem cells (HSCs) with the additional capability of self-renewal [35]. This discovery opened a gateway to treat leukemia, myeloma, and lymphoma through bone marrow transplants [31]. Understanding the potential of these multipotent stem cells leveled up the experiments conducted by the scientific world to explore better treatment options in other diseases. One such study was conducted in mouse models where mesenchymal stem cells that have the ability to form bones and cartilages were used in developing whole joints [26].

Induced pluripotent stem cells

**Figure 3:**
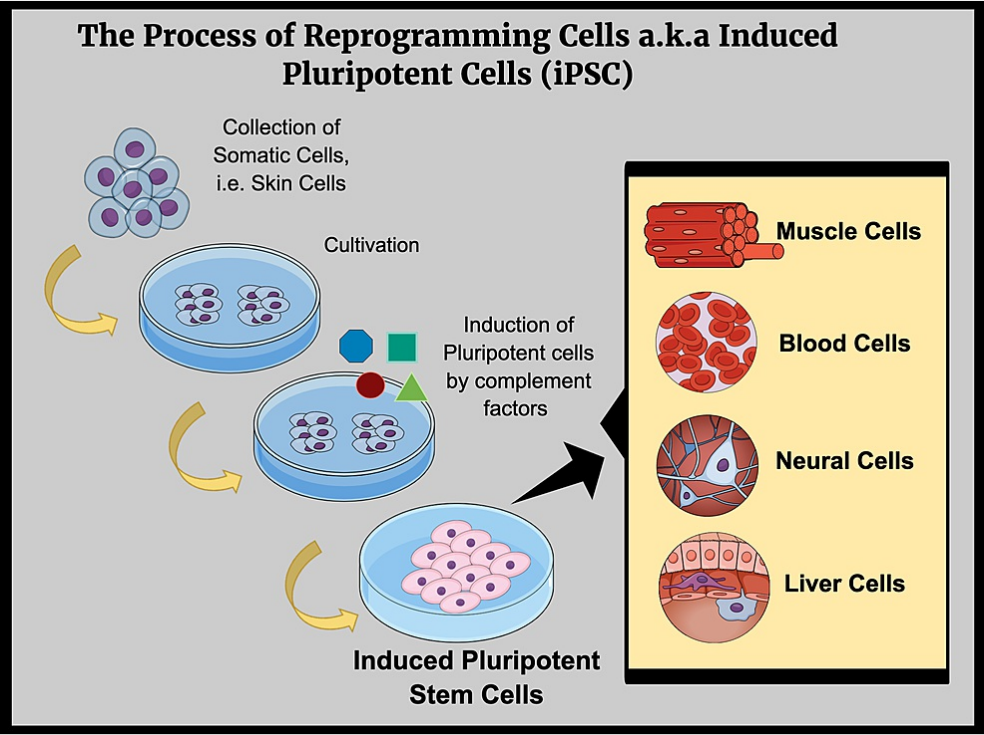
The Technique of Inducing Pluripotent Stem Cells Figure created in Mind the Graph platform

In order to use embryonic stem cells, in vitro fertilization occurs, after which these cells are extracted from the human embryos [36]. There have been numerous ethical conundrums in the process of deriving early stem cells. In 2006, scientists Yamanaka and Takahashi further investigated the concept of reprogramming a multipotent adult cell to a pluripotent cell, giving rise to a new type of stem cell known as induced pluripotent stem cells (iPSCs) [37]. The transformation of fibroblasts to a pluripotent state was possible due to four transcription factors, namely octamer-binding transcription factor 3/4 (Oct-3/4), sex-determining region Y-box 2 (Sox2), Kruppel-like factor 4 (KLF4), and cancer-related Myc gene [37]. Figure 3 depicts the process of cellular reprogramming involved in inducing pluripotent stem cells [30].

The discovery of iPSC pioneered in stem cell therapy research where humans could benefit from the customizability property and the biocompatibility aspect [37]. In short, this technique protects an individual from immune reactions and the adverse effects seen with multiple immunosuppressant agents that would be prescribed if embryonic stem cells were used. However, researchers are currently focused on minimizing the effects of tumorigenesis that may be evidenced in the process of reprogramming cells [31].

Modern medicine has been exploring and conducting numerous researches in a wide range of disorders to implement stem cells therapy starting from neurological disorders, cardiovascular disorders, bone disorders, liver diseases, radiation-induced damages, diabetes, asthma, etc. [38]. Larijani et al. studied stem cell therapy in various disorders and concluded that stem cells could be helpful in the control of immune-based illnesses owing to their immunomodulatory properties and stem cells' capability to differentiate into other cells, thus making headway possibly to an ultramodern therapy for organ or tissue dysfunction in the near future [38].

An overview of artificial intelligence in healthcare

Medical sciences' acceptance of technology is not a new concept, as AI has been employed in virtual and physical ways in today's healthcare. The virtual branch encompasses informatics techniques such as deep learning information to assess electronic health records and contributing physicians while counseling for various treatment options [39]. The physical aspect primarily reflects the robots employed to aid elderly patients and assist surgeons during procedures, including the emergence of a novel drug delivery system using targeted nanorobots [38]. Leonardo Da Vinci's preliminary sketches of robots helped set the platform for this innovation [4]. The legacy left behind by Da Vinci has progressed into today's thriving use of robotic-assisted surgery for performing complex urologic and gynecologic procedures [4].

Apart from robotics, a recent study by the International Business Machines Corporation (IBM) research team developed a new algorithm that combined machine learning and deep learning to diagnose breast cancer at an early stage [40]. They used data pertaining to mammography images, comprehensive clinical data, and biomarkers, thereby predicting the development of breast cancer in 87% of the cases examined, matching the precision to radiologists, and significantly minimizing the number of missed breast cancer diagnoses [40].

**Figure 4:**
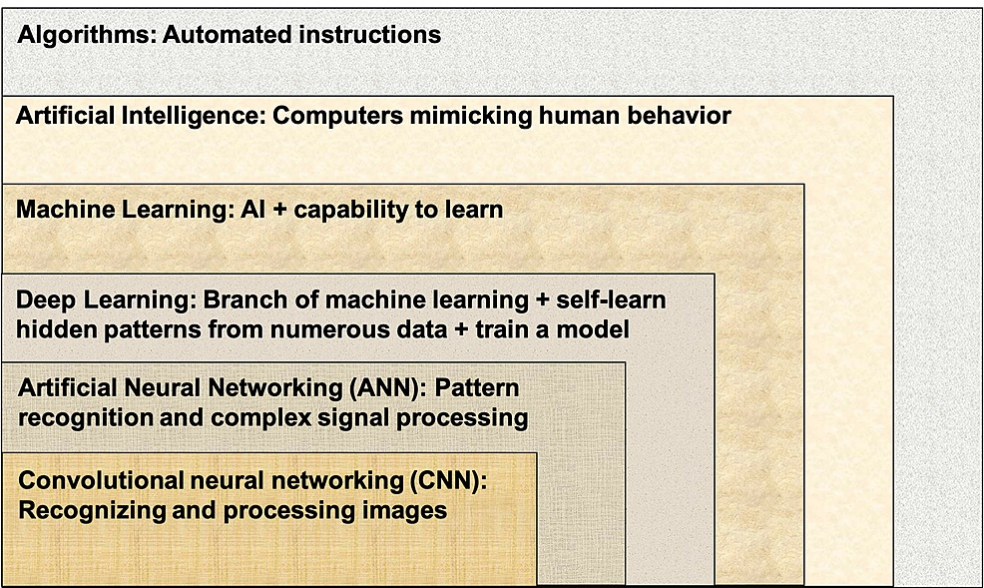
Hierarchy of Artificial Intelligence

The ultimate purpose of these medical technologies is to employ computer algorithms to extract useful information from data and aid clinical decision-making [38]. In summary, AI may help establish a diagnosis, select medication, formulate risks/benefits, and stratify illness while minimizing medical errors, thus enhancing productivity [39]. A highly specialized, single-purpose supercomputing Neural Network trainer can help generate the "Algorithms" without human intervention [39]. Figure 4 depicts the hierarchy of artificial intelligence commonly used in medicine [41].

**Figure 5:**
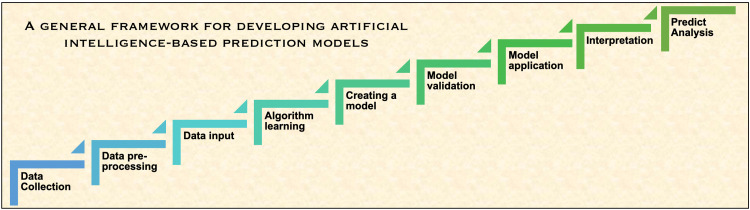
Framework for Developing AI-Based Prediction Models

The widespread availability of Graphics Processor Units opens the door to enhanced computing power, making parallel processing a high-speed entity with on-demand limitless storage capacity [39]. It is established that learning and interacting with more and more training data and algorithms allows additional insights into diagnostics, treatment options, and patient outcomes [42]. Figure 5 depicts the steps in creating a prediction model based on artificial intelligence [43].

According to researchers, AI is expected to substantially influence various areas of health care, including chronic illness management and clinical decision-making [39]. Studies to date have established few benefits in applying AI in healthcare and concluded that AI has the ability to: improve accessibility, diagnose early, minimize expenditures, assist in surgical procedures with efficiency, enhance human skills, and promote mental health support [44]. Apart from the benefits witnessed in this technology, there may be apparent flaws and risks, including injuries, privacy issues, inequality, discrimination, and professional restructure [44].

Application of artificial intelligence in stem cell therapy

**Figure 6:**
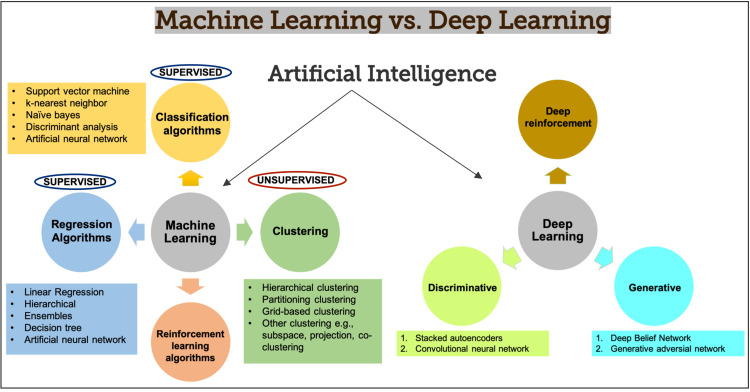
Difference in machine learning versus deep learning

Although stem cell therapy appears to be simple in theory, it is extremely difficult to describe all cell products since the cells are not stable or homogeneous, and existing testing methods may have more errors than predicted [21]. The possibility of slips when measuring something that could possess a million parameters is immense. Scientists believe artificial intelligence in various mediums such as data mining (DM), ML- SVM, and DL - CNN could help provide accurate measurements in solving this complexity, which could be the key to perfecting the formula for stem cell therapy [21]. An extensive analysis was done in the selected studies to deduce the relationship between AI and regenerative medicine. Figure 6 illustrates some of the most extensively used machine and deep learning algorithms [43] in medicine.

The study was done by Schaub et al. in collaboration with the National Eye Institute while attempting to speed up the quality assurance measurements of the stem cells in treating age-related Macular degeneration (AMD). AMD is an eye disorder where people lose their eyesight due to aging. With DNN, the researchers predicted cell functions in different scenarios and settings from annotated images of cells. The AI program used a well-known model architecture, GoogLeNet [21]. Through this technology, images of lab-grown eye tissues were identified either as viable or non-viable. The AI only identified one wrong prediction out of the 36 predictions used to test this machine [21]. After complete training, the AI program could classify eye tissues more precisely and faster than any human. This technique of identifying viable stem cells is called QBAM [21]. Numerous features of the cells, donor identity, and the outlier sample could be identified on the implanted tissue with an automated brightfield microscope within minutes without the need for clinical expertise [21]. This non-invasive testing method could minimize errors and prevent undesirable adverse effects in the field of stem cell therapy.

Fan et al. study derived iPSCs from human urinal cells and used CNN for colony recognition and semi-supervised segmentation, an essential aspect of machine learning that can understand visual information from limited labeled data to detect colony location and boundaries. During the reprogramming process of cells, this study used the Hidden Markov model (HMM) in order to predict the growth phase and maturation time window of colony formation [18]. HMM is an effective learning algorithm that can utilize raw data without preprocessing [45], and it is widely used in image processing, where the observations are a sequence of images in time [46]. The mouse-derived iPSC and urinal cells iPSC were verified using a computer vision algorithm that incorporated both a generated binary image and a brightfield image, including the colony-picking decisions, which were then cross-referenced manually [18]. The results were then compared to human embryonic stem cells. The computer vision approach significantly concluded that it worked for both human iPSC detection and the mouse model [18].

Shouval et al. applied the ML method to clinically predict overall mortality at 100 days after all-HSCT using data mining. One of the ML algorithms, the alternating decision tree (ADT) algorithm, was used in order to calculate using independent variables (as first-level decision nodes) and dependent variables (as daughter decision nodes) [17]. The independent variables included: stages of the disease, Karnoisky performance score, donor type, recipient donor serostatus, and HSCT year, while the dependent variables included: age, diagnosis, days from diagnosis to transplantation, conditioning regimen, and annual no of transplantation [17]. The sum of all the predictive values along all paths served as the continuous probabilistic means for classification as either positive or negative as well as higher or lower values following which patient was classified in the corresponding group [17].

Juhola et al. used six cell lines of catecholaminergic polymorphic ventricular tachycardia (CPVT)-specific iPSC-cardiomyocytes that consisted of 128 calcium transient signals for each baseline, adrenaline, and dantrolene (a muscle relaxant drug) conditions. Three measurements were recorded from human iPSC cardiomyocytes' calcium transient data via the machine learning method [23]. First, transient signal beats were identified using the peak recognition algorithm, then 12 peak variables were computed for each identified peak of a signal, and using this data, signals were classified into different classes corresponding to those affected by adrenaline or, later by dantrolene which showed calcium abnormalities during adrenaline perfusion [23]. The results were classified as a baseline condition, adrenaline condition, and dantrolene condition. The dantrolene condition was further sub-classified depending on the percentage of abnormalities reduced by dantrolene while reflecting the shape of the calcium peaks as either responder, semi-responder, or non-responder [23]. The following algorithms were used: k-nearest neighbors (KNN), Mahalanobis discriminant analysis (MDA), classification and regression trees (CART), multinominal logistic regression, Naïve Bayes, random forests, and least square support vector machine (LS-SVM), among which random forests and LS-SVM showed the highest accuracy [23].

The newly suggested framework for integrating image processing methods with the V-CNN model yielded promising results in identifying the quality of iPSC colonies in the study by Kavita et al. [19]. They selected elements of colony morphology and textures from the segmented colony and entered these inputs into the V-CNN model, making it the first time for input feature vector classification for colony quality using CNN. The selected characteristics were added into the V-CNN model to discriminate between healthy and unhealthy iPSC colonies to examine classification performance [19]. The V-CNN model's applicability for handling the classification job demonstrates greater classification accuracy than the competing SVM classifier. The experimental findings showed that the suggested deep V-CNN technique could detect colony quality with a 95.5% accuracy, surpassing the SVM classifier - with an accuracy of 75.2%, and hence, be a viable decision support model for clinical applications [19].

CGAN was used in the deep-learning framework to develop AI models for segmenting cancer stem cells in cultures and tumors in order to discover undescribed morphological traits in these cells in the study by Aida et al. [22]. AI was trained using fluorescence images of the Nanog-Green fluorescent protein, which was shown to be expressed in CSCs and phase-contrast images. Several parameters for assessing segmentation quality increased as a result of image selection for training, resulting in a unique approach for detecting the presence of Nanog-expressing cells in cultures and tumors. The AI established in this work was not as effective as green fluorescent protein (GFP) fluorescence analysis in recognizing Nanog-expressing cells; nonetheless, it might be improved for AI-aided diagnostic techniques of CSCs [22]. Concurrently, CNN could identify cell shape, nucleus, mitosis, and hemorrhage. The presence of mouse embryonic fibroblasts feeder cells resulted in the highest image assessment results. The AI model was seen to visualize CSCs in terms of GFP fluorescence using phase-contrast images [22].

Waisman et al. aimed to distinguish pluripotent stem cells from early differentiating cells using CNN, trained with transmitted light microscopy images. A total of 1,116 images were analyzed with two networks: the Dense Convolutional Network (DenseNet) with simple augmentation (DenseNet-SA) and with Residual Network 50 (ResNet50) with none or simple image augmentation (ResNet-SA) [20]. In a nutshell, a DenseNet is a type of convolutional neural network that employs dense connections between layers via Dense Blocks, where all layers are connected (with matching feature-map sizes) directly with each other and maintain the feed-forward nature, where each layer obtains additional inputs from all preceding layers and passes on its feature-maps to all subsequent layers [47]. ResNet is a type of artificial neural network that is based on an architecture known from pyramidal cells in the cerebral cortex [48], where additional layers are added to deep neural networks to enhance accuracy and performance, which is effective in addressing complicated problems [49]. ResNet does this by using skip connections, or shortcuts, to leap over some levels [48]. In this study, confusion matrices demonstrated that both neural networks predicted higher accuracy than the differentiating group; however, some variability was noted, particularly with DenseNet. The neural networks were extremely sensitive to morphological alterations; nevertheless, the changes were modest, including only minor alterations on the cell surface. Other benefits of using a neural network to cell models include continuous, automated, real-time detection with great precision [20]. However, it was discovered that excessive image preprocessing was unfavorable to accuracy and loss, and it was concluded that limited flipping of images in both directions could bring out beneficial training. The trained CNN recognized PSCs from very early, distinguishing PSCs with a very high prediction rate, almost close to one [20].

One other important concern for stem cell therapy is biosafety and bioefficacy. Zaman et al. [24] reviewed this aspect by taking advantage of machine learning, where the use of imaging data and analysis characterized morphological and phenotypic changes in stem cells by comparing data from cancer and stem cells under various conditions and environmental perturbations, as well as coupling it with deep learning algorithms like CNN, SVM, and Naïve Bayes, which could ensure biosafety and bioefficacy [24]. Table 3 below summarizes the algorithms used in studies chosen for this review.

**Table 3:** Implementation of artificial intelligence in various ways iPSC: induced pluripotent stem cells; ML: machine learning; V-CNN: vector-based convolutional neural network; SVM: support vector machine; CNN: convolutional neural network; iPSC-RPE: induced pluripotent stem cells-retinal pigment epithelial; DNN: deep neural network; QBAM: quantitative brightfield absorbance microscopy; DL: deep learning

Study	Type of stem cell studied	Type of AI used
Shouval et al. [17]	Allogeneic hematopoietic stem-cell	DM, ML - alternating decision tree
Fan et al. [18]	iPSC	ML
Kavita et al. [19]	iPSC	V-CNN and SVM
Waisman et al. [20]	Mouse embryonic stem cells	CNN
Schaub et al. [21]	iPSC-RPE	DNN and QBAM
Aida et al. [22]	Cancer stem cells	DL and CGAN
Juhola et al. [23]	iPSC Cardiomyocytes	ML
Zaman et al. [24]	All types of stem cells	ML and DL

Limitations

This study mainly focused on various aspects of AI application in stem cell therapy, from pre-treatment to post-treatment. An overview of AI in stem cell therapy is discussed, and a pinpoint analysis of specific subtypes was not done in detail. Studies after 2015 were only analyzed to understand the recent advancements in a diverse ethnic population, including animal studies. However, substantial biosafety and bioefficacy studies must be conducted before and after treatment, including targeted human trials in which the disease status and control groups are labeled while also considering any ethical eminence.

## Conclusions

Creating a general-purpose "high-performance computing platform" is becoming a reality in creating highly specialized systems to enhance regenerative medicine, as evidenced by the studies in this review. It is possible to conclude that AI plays a crucial role in stem cell therapy in many ways, whether before, during, or after treatment, demonstrating the advancement of modern science. Algorithms, machine learning, data mining, deep neural network, and other subtypes mentioned in the included studies have stated that artificial intelligence could improve accuracy. These technological advancements have aided in detecting a reliable framework for iPSC colony classification, cellular morphology identification, noninvasive cell therapy characterization into healthy vs. unhealthy cells, identifying undescribed morphological traits in cancer stem cells, and precisely predicting drug effects. A user-friendly online tool incorporating the data mining methods for assessing risk in acute leukemic patients before administering hematopoietic stem cell therapy was also recognized as a fail-safe design. The convolutional neural network, the availability of vast medical images, and this specialized computing system training take machine intelligence to the next level. However, since it is in a primitive stage, as evidenced by limited studies so far, more research needs to be conducted to conclude the detailed benefits and risks concerning specific ailments associated with AI and stem cell therapy.
